# Hoarseness as Presenting Complain of a Glomus Vagale

**DOI:** 10.5812/kowsar.20741804.2248

**Published:** 2011-09-15

**Authors:** M Mozafar, H Molaei, M Hassani, M A Shahabedin

**Affiliations:** 1 Department of Vascular Surgery, Shohada-E-Tajrish Hospital, Shahid Beheshti University of Medical Sciences, Tehran, Iran

**Keywords:** Glomus vagal tumor, Paraganglioma, Hoarseness

Dear Editor,

Vagal paragangliomas are uncommon tumors of the parapharyngeal space, approximately 200 cases having been reported.[1] They arise from tissue of the extra adrenal sympathetic nervous system.[2] They mostly manifest as a painless neck mass near the angle of the mandible usually between the skull base and the hyoid bone.[3]

The other manifestations are hoarseness and less commonly dysphagia, palatal weakness and tongue hemi-atrophy.[1][4] Pressure on the cervical sympathetic chain causes horner syndrome. Ultrasonography, CT scan, MRI and angiography are diagnostic modalities been used, but combining conventional MR imaging with CE-MRA has dramatic diagnostic value for the assessment of head and neck paragangliomas.[5] Surgical resection is the treatment for carotid body tumors, and the procedure should be undertaken unless the patient has a prohibitive operative risk.[4] Here we present a rare glomus vagale case presented with hoarseness and show his management.

**Fig. 1:**
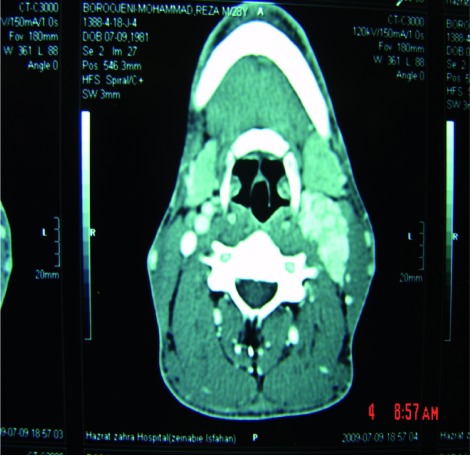
CT scan shows left neck mass.

A 37 years old farmer presented with hoarseness and left cervical mass beyond mandible angle. He did not claim of another symptom, on physical examination a firm mobile 2x2 cm mass was moving horizontally. Before treatment planning, we found left vocal cords paralyzed and CT scan demonstrated a heterogenous lesion next to the carotid bifurcation (Figure 1). Planning resection, he underwent surgery and we found two masses, one in 1.5x1.5 over bifurcation and the other just in bifurcation area extending toward skull through vagal foramen, we could resect these highly vascular masses while saving carotid branches but sacrificing involved vagal nerve. Pathologist confirmed these paragangliomas.

The normal carotid body is a small mass of neurovascular tissue located bilaterally in the medial valley of the carotid artery bifurcation. Although the term "tumor" has historically been assigned to these masses, they are not carcinomas, and the neoplastic process is actually hypertrophy of the carotid body tissue.[6]

The four main locations of glomus tissue within the head and neck are: (a) The carotid bifurcation (carotid body tumor), (b) The inferior ganglion region (ganglion nodosum) and cervical portion of the vagus nerve (glomus vagale or vagal paraganglioma), (c) The jugular bulb region (glomus jugulare) and (d) The middle ear cavity (glomus tympanicum).[3] Paragangliomas originate in the head and neck from the brachiomeric family of paraganglia, neuroendocrine tissue which lies along the carotid artery, the aorta, the glossopharyngeal nerve, and the middle ear.[7] Preoperative planning requires a selective carotid arteriogram to clarify arterial anatomy and the relation of the carotid body tumor to the carotid bifurcation. Embolization of the tumor may also be achieved through these branch vessels, particularly in cases in which the lesion extends over a considerable distance and may approach the base of the skull.

Challenging options about surgical versus radiation treatment continues, advocating radiation for small lesions,[8][9] and surgery in younger patients and tumors causing bony destruction.[10] Morbidity associated with vagal paraganglioma surgery is significantly higher than that of carotid body tumor surgery. Plans for intraoperative blood cell salvage are appropriate, as significant blood loss is routine in the dissection of these lesions.[6] Therapeutic embolization reduces the size of the tumor and limits intraoperative blood loss but may rarely be considered as definitive treatment.[11] Radiation therapy is an alternative to surgery and may be effective in arresting the growth, but it can lead to neurologic sequelae and rarely can destroy the tumor. Irradiation is generally used in elderly patients as well as in large and unresectable tumors [1] and gathering data upon clinical examination and imaging, we prepared our client to surgery. Under general anesthesia via standard cervical approach, tumor dissected off the carotid branches but found left vagus nerve involved, extending toward skull base through cranial nerve foramen, so had to sacrify it.
